# Supplementary material for: MetaRibo-Seq measures translation in microbiomes
Source: Nat Commun. 2020 Jun 29;11:3268. doi: 10.1038/s41467-020-17081-z (PMC7324362; doi:10.1038/s41467-020-17081-z)
Supplement: Supplementary file 10 — Supplementary Data 7 [file 41467_2020_17081_MOESM10_ESM.zip › File2/Confidence_High_Taxonomy/333550_out.krona.html]

Javascript must be enabled to view this page.

members
magnitude
magnitudeUnassigned
count
unassigned
taxon
rank

333550\_out

11

11
superkingdom
2

976
phylum
11

200643
class
11

171549
order
11

11
family
171552

838
genus
11


SRS013687\_contig\_number\_18811SRS016541\_contig\_number\_15259SRS017307\_contig\_number\_18196SRS023914\_contig\_number\_20703SRS043667\_contig\_number\_1674SRS1041031\_contig\_number\_30180SRS1041140\_contig\_number\_10526
7
species
165179

2293125
species
4

SRS012849\_contig\_number\_36496SRS013521\_contig\_number\_contig-100\_137.72613SRS049995\_contig\_number\_36404SRS078176\_contig\_number\_contig-100\_2187.114543
